# Fatigue following mild traumatic brain injury relates to visual processing and effort perception in the context of motor performance

**DOI:** 10.1016/j.nicl.2021.102783

**Published:** 2021-08-13

**Authors:** Roeland F. Prak, Jan-Bernard C. Marsman, Remco Renken, Joukje van der Naalt, Inge Zijdewind

**Affiliations:** a Department of Biomedical Sciences of Cells and Systems, University Medical Center Groningen, University of Groningen, Groningen, the Netherlands; b Department of Neurology, University Medical Center Groningen, University of Groningen, Groningen, the Netherlands

**Keywords:** mTBI, BOLD fMRI, Fatigability, FSS, MFIS

## Abstract

**Introduction:**

Following mild traumatic brain injury (mTBI), a substantial number of patients experience disabling fatigue for months after the initial injury. To date, the underlying mechanisms of fatigue remain unclear. Recently, it was shown that mTBI patients with persistent fatigue do not demonstrate increased performance fatigability (i.e., objective performance decline) during a sustained motor task. However, it is not known whether the neural activation required to sustain this performance is altered after mTBI.

**Methods:**

Blood oxygen level-dependent (BOLD) fMRI data were acquired from 19 mTBI patients (>3 months post-injury) and 19 control participants during two motor tasks. Force was recorded from the index finger abductors of both hands during submaximal contractions and a 2-minute maximal voluntary contraction (MVC) with the right hand. Voluntary muscle activation (i.e., CNS drive) was indexed during the sustained MVC using peripheral nerve stimulation. Fatigue was quantified using the Fatigue Severity Scale (FSS) and Modified Fatigue Impact Scale (MFIS). Questionnaire, task, and BOLD data were compared across groups, and linear regression was used to evaluate the relationship between BOLD-activity and fatigue in the mTBI group.

**Results:**

The mTBI patients reported significantly higher levels of fatigue (FSS: 5.3 vs. 2.6, p < 0.001). Both mTBI- and control groups demonstrated significant performance fatigability during the sustained MVC, but no significant differences in task performance or BOLD-activity were observed between groups. However, mTBI patients reporting higher FSS scores showed increased BOLD-activity in the bilateral visual cortices (mainly extrastriate) and the left midcingulate gyrus. Furthermore, across all participants mean voluntary muscle activation during the sustained MVC correlated with long lasting post-contraction BOLD-activation in the right insula and midcingulate cortex.

**Conclusion:**

The fMRI findings suggest that self-reported fatigue in mTBI may relate to visual processing and effort perception. Long lasting activation associated with high levels of CNS drive might be related to changes in cortical homeostasis in the context of high effort.

## Highlights

• Post-mTBI fatigue relates to visual processing and effort perception.
• Fatigue correlates with activity in the extrastriate cortex and left midcingulate.
• CNS drive during a sustained effort correlates with post-contraction BOLD-activity.
• Insular and midcingulate activation suggests changes in cortical homeostasis.

## 1 Introduction

Traumatic brain injury is a major health issue, affecting (healthy) adults across all age groups (Bazarian et al., 2005, Cassidy et al., 2004). Most injuries are closed head injuries, in which there is rapid acceleration/deceleration of the brain which may lead to (diffuse) axonal injury (Armstrong et al., 2016, Bazarian et al., 2005). The majority of traumatic brain injuries presenting to hospitals are classified as mild (mTBI) and although most of these patients recover, a substantial number of patients suffer from persistent symptoms (Cassidy et al., 2004, Levin and Diaz-Arrastia, 2015, McMahon et al., 2014). Patients may experience cognitive problems – impairment of memory, attention, and executive functions – as well as affective complaints (Carroll et al., 2014, Lundin et al., 2006). One of the most frequent complaints following mTBI is fatigue (Lundin et al., 2006, Norrie et al., 2010, van der Naalt et al., 2017). Fatigue limits individuals in their activities, has a negative impact on quality of life, and may impede return to work (de Koning et al., 2017, Stulemeijer et al., 2006, Wäljas et al., 2012). To date, an effective treatment for fatigue is lacking; as is the understanding of its underlying mechanisms.

Fatigue is a symptom that is measured by self-report, and is thought to be mediated by performance fatigability and perceived fatigability (Enoka and Duchateau, 2016, Kluger et al., 2013). Performance fatigability is defined as an objective decline in performance, which can be assessed during motor or cognitive tasks. Since executive functioning and attentional capacity are often affected by mTBI, most studies in mTBI have utilized cognitive tasks to assess performance fatigability. However, only few studies have focussed on changes in neural activation in relation to performance fatigability, either during (Liu et al., 2016, Möller et al., 2017, Wylie et al., 2017) or after (Nordin et al., 2016) a sustained cognitive task, and with varying results. Drawbacks of utilising cognitive tasks to study performance fatigability include difficulty in monitoring learning effects that may confound performance decline (Möller et al., 2019). These issues may be overcome by utilising a motor task, as monitoring force output directly reflects output of the central nervous system and provides relatively straightforward means to index performance fatigability.

In this study, we follow up on recent findings that mTBI patients with persistent fatigue (>3 months) do not demonstrate increased performance fatigability during a sustained motor task (Prak et al., 2019). We used blood oxygen level-dependent (BOLD) imaging to assess whether the neural activity necessary to maintain this level of performance is similar in patients as compared to healthy controls, or whether patients have to increase their activity in order to compensate for mTBI-related changes in CNS integrity. A secondary aim of the present study was to identify potential neural substrates of fatigue, by exploring the relationship between self-reported fatigue (indexed using questionnaires) and BOLD activity.

## 2 Materials and methods

### 2.1 Study population

Twenty patients with mTBI (7 females, age: 23–57, 2 left-handed), defined by a Glasgow Coma Scale score between 13 and 15 on admission, posttraumatic amnesia < 24 h, and/or loss of consciousness < 30 min, were included in the study. All mTBI patients had persistent complaints of fatigue (>3 months post injury). Twenty control participants were included and matched with the mTBI group for age and sex (7 females, age: 21–59, none left-handed). Exclusion criteria included psychiatric disorders, neurologic disease (including previous TBI), drug or alcohol abuse, and contraindications for MRI. The experimental procedures were in accordance with the Declaration of Helsinki (World Medical Association, 2013) and were approved by the medical ethical committee of the University Medical Center Groningen. All participants provided written informed consent.

### 2.2 Questionnaires and cognitive tests

Self-reported fatigue was quantified using the Fatigue Severity Scale (FSS; Krupp et al., 1989) and Modified Fatigue Impact Scale (MFIS; MS Council for Clinical Practice Guidelines, 1998). Depressed mood was evaluated using the Hospital Anxiety and Depression Scale (HADS; Zigmond and Snaith, 1983). The Symbol Digit Modalities Test (SDMT; Smith, 1982) and the 3 s Paced Auditory Serial Addition Test (PASAT’3; Gronwall, 1977, McCauley et al., 2014) were used to evaluate cognitive impairment and attentional processing. Handedness was measured using the Edinburgh inventory (Oldfield, 1971).

### 2.3 Force recording

**Fig. 1:**
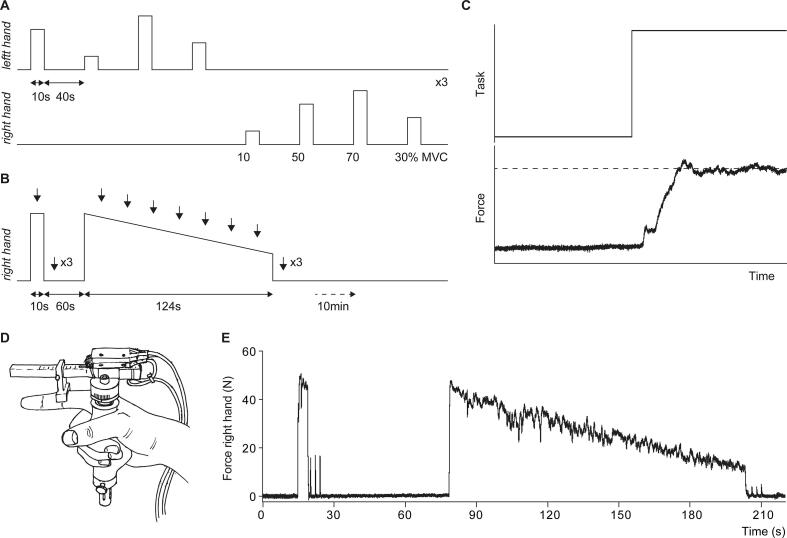
**Experimental paradigm and raw data.***Schematic illustration of the experimental paradigm for (A) the submaximal contraction task and (B) the sustained MVC. (C) Visual feedback of the task and the produced force data provided to participants, note the horizontal cursor which was used to indicate the target force during the submaximal contractions. (D) Illustration of the force transducer. (E) Raw force data recorded from a mTBI participant during the sustained MVC task.*

Index finger abduction force was recorded from both hands using MR-compatible force transducers (Fig. 1D; Duinen et al., 2007). The horizontal bar of the transducer was aligned parallel to the index finger, the index finger was extended and the finger bracket was positioned over the proximal interphalangeal joint. To maintain this position throughout the experimental session, the transducers were taped to the participant’s hands. Force signals were sampled at 500 Hz using a 1401 micro interface and Spike2 software (version 7.12, Cambridge Electronic Design, Cambridge, UK).

### 2.4 Muscle activation

Voluntary muscle activation of the right FDI was determined using the interpolated twitch technique (Gandevia, 2001, Merton, 1954). The ulnar nerve was stimulated at the right wrist using a constant-current stimulator placed outside the scanner room (DS7A, Digitimer, Welwyn Garden City, United Kingdom), and stimulating electrodes entered the scanner room via a radio frequency wave-guide. Doublet forces were evoked using paired pulses (10 ms interval) to increase the signal-to-noise ratio (Gandevia and McKenzie, 1988), and pulse width was set to 500 µs to adjust for the filtering characteristics of the wave-guide (Post et al., 2009). Stimulation intensity was determined by increasing the stimulator output by 5 mA increments until a maximal force response was obtained.

### 2.5 MRI acquisition

Scans were acquired using a 3 Tesla Philips Intera Achieva MRI scanner (Philips Medical Systems, Best, the Netherlands) equipped with a 32-channel SENSE head coil. Functional images were obtained using an echo-planar imaging sequence (echo time = 29 ms; repetition time = 2 s; flip angle = 82°; 39 slices; 3.5 mm slice thickness; field of view 224 mm; matrix size 64 × 64; transverse slice orientation). A T1-weighted anatomical scan was obtained for co-registration of the functional images (echo time = 30 ms; repetition time = 9 ms; flip angle = 8°; 170 slices; 1 mm slice thickness; field of view = 256 mm; matrix size 256 × 256; transverse slice orientation).

### 2.6 Motor tasks

The protocol and set-up were similar to earlier experiments in control participants (Post et al., 2009) and persons with multiple sclerosis (Steens et al., 2012). Each participant was familiarised with the tasks during a training session that took place approximately one week prior to the scanning session. During the MR session, participants lay supine in the scanner with their arms extended alongside them and a force transducer in each hand. Their head was immobilised using foam padding. Via a mirror on top of the head coil, participants could see a screen displaying their own force level and signals indicating when to start and end each contraction. The scanning session consisted of three tasks:

*Task 1*. Participants generated six maximum voluntary contractions (MVCs) with their index finger abductor, alternating between the left and right hand (3 per hand). Contractions were sustained for 10 s with 50 s rest between contractions.

*Task 2*. Participants performed six blocks of contractions (3 per hand) at 10, 30, 50, and 70% MVC (Fig. 1A). Participants were instructed to match their force level to a horizontal cursor indicating the target level (Fig. 1C). Each contraction was sustained for 10 s with 40 s rest between contractions.

*Task 3*. Participants performed a brief MVC (6 s) with their right hand followed by 60 s rest and a sustained MVC (124 s; Fig. 1B). To quantify voluntary muscle activation during the sustained contraction, the ulnar nerve was stimulated (paired-pulses) and superimposed doublet forces were evoked during the brief MVC and at 7 time-points during the sustained MVC (18 s interval). Doublet forces were also evoked at rest (2 s interval) after both the brief MVC (i.e., initial-doublet) and the sustained contraction (i.e., post-doublet). After the sustained MVC, scanning continued for 9 min. During all tasks participants were instructed to keep their eyes open.

### 2.7 Analysis: Questionnaires and task data

Questionnaire and cognitive test scores were calculated. Maximum force was determined for the MVCs in task 1. For the submaximal contractions (task 2), mean force during the plateau phase of each contraction was calculated (2 s after the start and 1 s before the end of each contraction) and expressed as percentage of MVC. For the sustained MVC (task 3), mean force was calculated over 2 s epochs. The mean force during the first (i.e., initial force) and last (i.e., residual force) 6 s of the sustained MVC were determined and expressed as percentage of MVC. The amplitudes of the evoked doublet forces were determined and expressed as percentage of the largest doublet at rest (i.e., initial-doublet). Superimposed doublets evoked during the sustained MVC were linearly corrected for the use-dependent decline in contractile function (conform: Prak et al., 2019, Schillings et al., 2003), and voluntary muscle activation was calculated as (1 – superimposed doublet/initial-doublet) × 100%. For all three tasks, start and end times of the muscle contractions were determined for use in the general linear models (GLMs).

Statistical analysis was performed in RStudio (R version 3.6.1). Data are presented as number (percentage), mean (SD), or median (range) for categorical, normally distributed, and non-normally distributed data, respectively. Differences between mTBI and control groups were assessed using mixed-model ANOVAs. Sex was included as covariate for MVC, initial- and post-doublet, and residual force to account for sex-related variance (Sars et al., 2018). Welch correction was used when variances between groups were unequal. Model residuals were inspected for normality using Q-Q plots and if required the dependent variable was transformed (Prescott, 2019). If normality could not be achieved, a Mann-Whitney *U* test was used (HADS). P-values < 0.05 were considered statistically significant.

Multilevel regression models were used to examine time-related changes in force and voluntary muscle activation over the course of the sustained MVC. The time-course was modelled by including fixed-effects of time, time^2^ and time^3^ in the model in a stepwise fashion. After each step, statistical analysis determined whether the more complex model survived (indicated by a decrease in the Akaike information criterion score ≥ 2). Model intercepts and slopes for time and its polynomials were allowed to vary randomly per participant. Next, we tested for fixed effects of group (mTBI), sex, and age, as well as interactions with time. Model residuals were examined graphically for normality and heteroscedasticity. If required, the dependent variable was transformed to meet these criteria (superimposed doublets). Finally, robustness of the final models was tested by re-estimating the model on a trimmed dataset. This was done by first identifying model outliers (data points with scaled residuals > 2), removing these data points from the dataset, and then re-estimating the model.

### 2.8 Analysis: BOLD data

MRI data were analysed using SPM12 (Wellcome Trust Centre for Neuroimaging, Institute of Neurology, London, UK, http://www.fil.ion.ucl.ac.uk/spm) implemented in MATLAB 8.5 (R2015a, The MathWorks, Inc., Natick MA, USA). Functional images were pre-processed by realigning to the mean image, co-registering to the T1-weighted anatomical image, and normalizing to the standard MNI template. Data were smoothed with a Gaussian kernel of 8 mm full width at half maximum (FWHM), and log-transformed.

GLMs were created for each experimental task. For task 2, contractions with the left and right hand were modelled as separate conditions; timing of the conditions (i.e., onset and duration) was based on the force data. The mean force during each contraction was included as parametric covariate. For task 3, the sustained contraction was divided into three intervals of even length (Int1, Int2, Int3) to examine both the effect of task (mean across all intervals) and time-related changes in activation (Int1 vs. Int3; conform Post et al., 2009). A two-minute interval after the end of the sustained MVC was modelled to account for post-contraction changes, and the time-points of the electrical stimulation were included as a separate condition. All conditions were convolved with the canonical hemodynamic response function. For task 3, a linear trend lasting the entire length of the run was included in the model to account for slow drift in the BOLD signal as no high pass filtering was applied. Realignment parameters, corresponding to the rigid body transformations applied during image pre-processing, were used to calculate the framewise displacement across scans. Motion-censoring was applied to images if the framewise displacement exceeded a threshold of 0.9 mm (Siegel et al., 2014) as part of the first level analysis. Additionally, participants with a root-mean-square of the motion parameters exceeding 1.5 mm (across an entire run) were excluded from the analysis (no occurrences). The realignment parameters were not included in the GLMs.

At second level, two-sample *t*-tests were performed to assess task-related activation relative to baseline and differences between the mTBI and control groups. For group comparisons, a cluster forming threshold of p < 0.001 (uncorrected) was used and only clusters with a p-value < 0.05 (family-wise error corrected) were considered statistically significant. Additionally, regression analysis was performed to assess associations between task-related changes in BOLD-activation in the mTBI group and scores on the cognitive tests and fatigue questionnaires. To normalize for variation in mood, HADS depression scores were included as regressor of no interest. All activation coordinates are reported in MNI space.

In addition to the two-sample *t*-tests, a supervised learning technique (SSM/PCA) was used to identify differences in (task related) BOLD-activation between the mTBI and control groups (Moeller and Strother, 1991, Spetsieris et al., 2009, Spetsieris and Eidelberg, 2011). In brief, a principal component analysis was performed over the first-level contrast images (per task). Participants received a score for each principal component. Next, logistic regression was performed on these scores (control versus mTBI), and a combination and weighting of the principal components was determined that could separate the two groups.

## 3 Results

One mTBI participant was excluded from the analysis due to artefacts in the MRI data. To maintain balanced groups, the age- and sex-matched control was also excluded. Demographics of the remaining participants, and clinical characteristics of the mTBI group are provided in Table 1.

**Table 1:** **Demographics and clinical characteristics**.

	mTBI *(n = 19)*	Control *(n = 19)*
**Demographics**		
Sex		
Male	13 (68.4%)	13 (68.4%)
Female	6 (31.6%)	6 (31.6%)
Mean age (years)	40.7 (23–57)	40.6 (21–59)
**Clinical characteristics**		
GCS on admission		
13	5 (26.3%)	
14	12 (63.2%)	
15	2 (10.5%)	
Mechanism of injury		
Fall	5 (26.3%)	
Traffic accident	9 (47.4%)	
Sports injury	4 (21.1%)	
Occupational injury	1 (5.3%)	
Median time post-injury (months)	5 (3–13)	
**Cognitive tests**		
SDMT raw score	51 (36–72)	
T-score ≤ 40	4 (21.1%)	
PASAT ‘3 (% correct)	83.3 (51.7–98.3)	
<5th percentile	1 (5.3%)	
**Questionnaire scores**		
FSS	5.3 (0.8)	2.6 (0.9)
MFIS physical	20 (5–32)	
MFIS cognitive	27 (16–35)	
HADS depression	7 (2–14)	0 (0–2)
HADS anxiety	6 (4–12)	4 (0–7)
Oldfield	90 (-100–100)	90 (50–100)

Data are presented as numbers (percentage), mean (standard deviation), or median (range). PASAT scores below the 5th percentile (corrected for education; Rao et al., 1991) or SDMT T-scores ≤ 40 (corrected for education and age; Smith, 1982) were indicative of impairment.

GCS = Glasgow Coma Scale; FSS = Fatigue Severity Scale; MFIS = Modified Fatigue Impact Scale; HADS = Hospital Anxiety and Depression Scale; PASAT = Paced Auditory Serial Addition Test; SDMT = Symbol Digit Modalities Test.

### 3.1 Fatigue questionnaires

The FSS scores indicated higher levels of self-reported fatigue in mTBI than control participants (5.3 ± 0.8 vs. 2.6 ± 0.9, p < 0.001). Eighteen mTBI participants scored above the cut-off of 4, indicating significant fatigue (Krupp et al., 1989), compared to none of the controls. Median scores for the MFIS questionnaire and cognitive tests are reported in Table 1. HADS depression scores were higher in mTBI participants (median: 7, 2–14 vs. 0, 0–2, p < 0.001).

### 3.2 Force

Physiological data are presented in Table 2. No differences in MVC or electrically evoked force at rest (i.e., initial-doublet) were observed across groups. All participants performed the submaximal contractions (task 2) as instructed; mean force at each target level did not differ between the groups (Table 2).

**Table 2:** **Physiological data**.

		mTBI *(n = 19)*	Control *(n = 19)*	p-value	
				Group	Sex
MVC left (N)	F	41.3 (7.5)	41.2 (5.5)	0.958	<0.001*
	M	54.7 (7.2)	54.5 (15.5)		
MVC right (N)	F	35.2 (6.7)	33.2 (7.1)	0.556	0.004*
	M	43.3 (7.3)	42.0 (9.2)		
Initial-doublet (N)	F	11.7 (3.2)	10.1 (3.6)	0.061	0.020*
	M	15.7 (4.2)	12.8 (4.2)		
Initial-doublet (%MVC)		35.6 (8.5)	31.8 (12.4)	0.280	0.498
**Submaximal contractions**					
10%	L	11.9 (1.6)	11.8 (2.3)	0.845	–
	R	12.0 (1.4)	11.7 (2.0)	0.500	–
30%	L	30.3 (1.5)	29.7 (3.4)	0.452	–
	R	30.3 (1.1)	29.7 (1.8)	0.303	–
50%	L	49.5 (1.6)	48.9 (1.9)	0.303	–
	R	49.3 (1.4)	49.1 (2.2)	0.794	–
70%	L	68.8 (2.1)	68.0 (2.7)	0.338	–
	R	68.9 (1.8)	67.9 (2.5)	0.198	–
**Sustained MVC**					
Initial force (%MVC)		83.2 (6.7)	81.3 (6.6)	0.394	–
Residual force (%MVC)	F	36.2 (3.9)	39.3 (4.8)	0.467	0.007*
	M	28.6 (7.8)	30.1 (11.4)		
Mean voluntary muscle activation (%)		85.0 (8.3)	84.6 (10.1)	0.913	–
Post-doublet (%Initial)		47.4 (18.4)	58.9 (22.6)	0.092	0.115

Data presented in the table are mean (standard deviation), or median (range).

*MVC = Maximal voluntary contraction; F = female; M = male; L = left; R = right.*

During the sustained contraction (task 3), two mTBI participants briefly stopped contracting at 2 and 3 time points for approximately 2–5 s. Force data during these epochs were excluded from the analysis. Over the course of the sustained MVC, force declined non-linearly as demonstrated by significant effects of time, time^2^, and time^3^ to the explanatory model. No effect of group was observed, but force declined more and more quickly in stronger participants, as indicated by the significant effect of MVC (t = −4.93, p < 0.001) and the interaction effect between MVC and time (t = −2.41, p = 0.021). Force also declined more in male participants (t = −2.10, p = 0.040).

### 3.3 Voluntary muscle activation

Superimposed doublets from one mTBI participant were missing due to a technical problem with the stimulation. Over the course of the sustained MVC, both groups demonstrated a decline in (square root transformed) voluntary muscle activation (t = -2.53, p = 0.016), indicating inability of the central nervous system to sustain optimal output to the muscle. However, no differences were observed between the groups. After the sustained MVC, the electrically evoked force at rest (i.e., post-doublet) declined to 47.4 ± 18.4% of the initial value in mTBI versus 58.9 ± 22.6% initial in controls (p = 0.092).

### 3.4 BOLD-activation: mTBI vs. Controls

A summary of the motion statistics and censoring is provided in the Appendix A, Eickhoff et al., 2005, Mayka et al., 2006 (Supplementary Table A and B). For the sustained MVC, data from five participants (3 mTBI) were excluded due to excessive head motion (i.e., <10 scans remaining per condition).

**Fig. 2:**
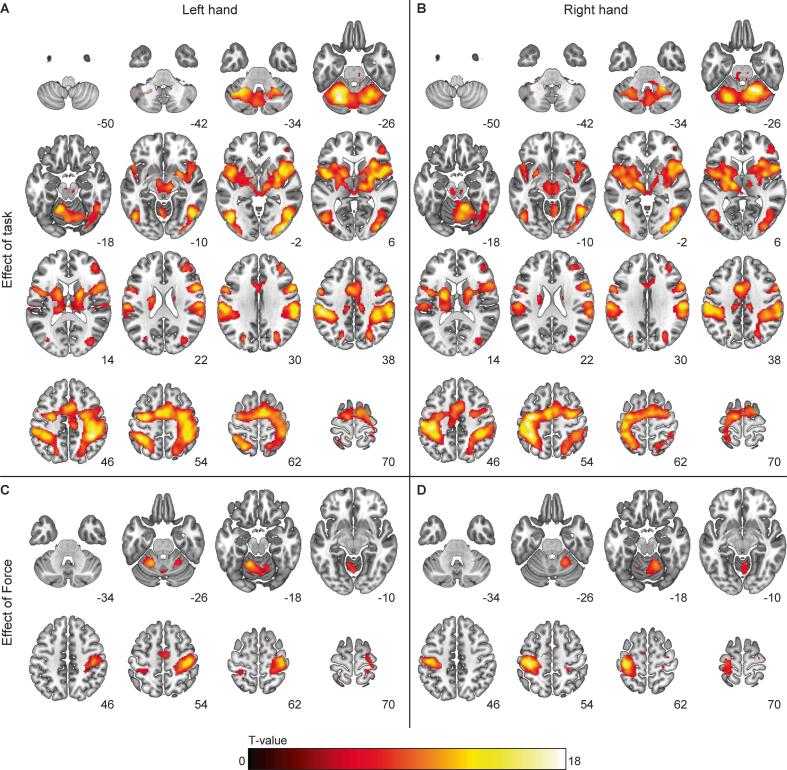
**BOLD-activation during the submaximal muscle contractions.***Axial slices showing BOLD-activation during the submaximal contractions for the effect of ‘task’ with (A) the left hand and (B) the right hand. The effect of ‘force’ is shown in the lower panels for (C) the left- and (B) the right hand, and shows activation in the contralateral sensorimotor cortex and ipsilateral cerebellum. BOLD data are shown for both groups combined. The colour bar indicates voxel T-values, all voxels shown are significant at p < 0.05 family-wise error corrected. Z coordinates (MNI space) are shown for each slice, left is left according to neurological convention.*

**Fig. 3:**
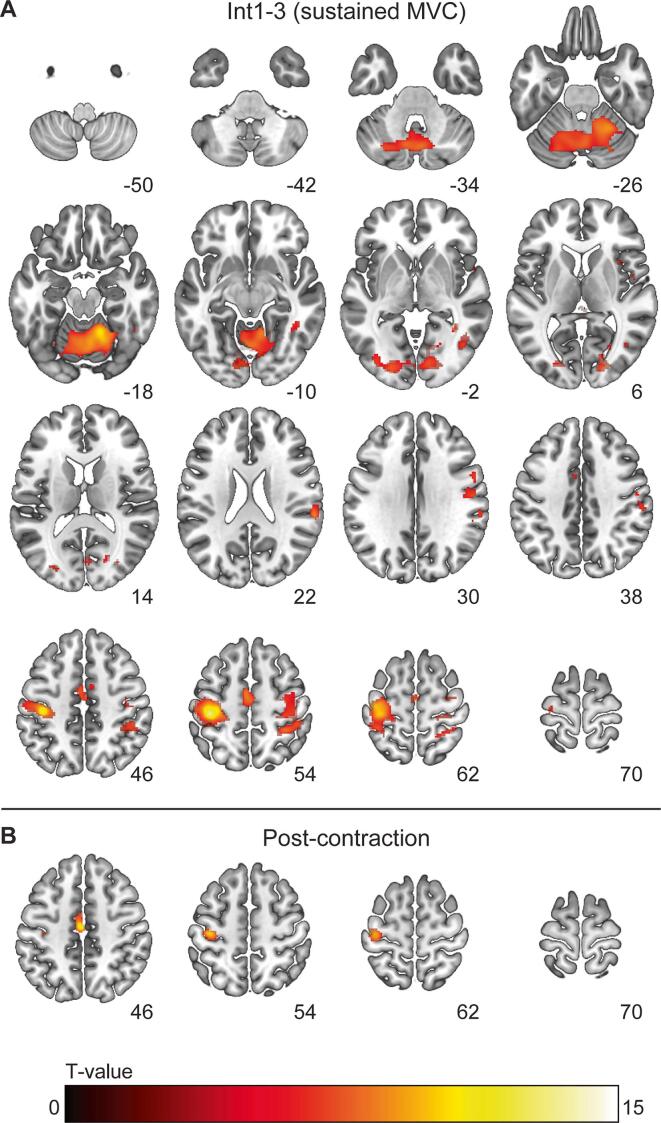
**Task and post-contraction activation for the sustained MVC.***Axial slices showing BOLD-activation (A) during the sustained contractions and (B) for the post-contraction activation. Data are shown for both groups combined. The colour bar indicates voxel T-values, all voxels shown are significant at p < 0.05 family-wise error corrected. Z coordinates (MNI space) are shown for each slice, left is left according to neurological convention.*

BOLD-activation during task 2 and 3 are shown in Fig. 2, Fig. 3, respectively. During the submaximal contractions, mean force level per contraction was positively associated with activation of the contralateral sensorimotor cortex and ipsilateral cerebellum, without differences between the groups. For the sustained MVC, an increase in BOLD-activation over the course of the sustained MVC (i.e., Int3 > Int1) was seen in a large cluster (1038 voxels) with peak activation in the left SMA extending to the left primary motor cortex (Supplementary Table C). Activation decreased (i.e., Int3 < Int1) in the left middle orbital gyrus and right cerebellum (lobes V-VI and vermis 4–5). No significant differences in BOLD-activation were observed between groups for the effect of task, post-contraction activation, or for the time-related changes in activation (Int1 vs. Int3). Similar to the results of the mass univariate analysis, the supervised learning technique (SSM-PCA) was unable to differentiate between mTBI and control groups across either of the experimental tasks.

### 3.5 BOLD regression (I): Voluntary muscle activation

**Fig. 4:**
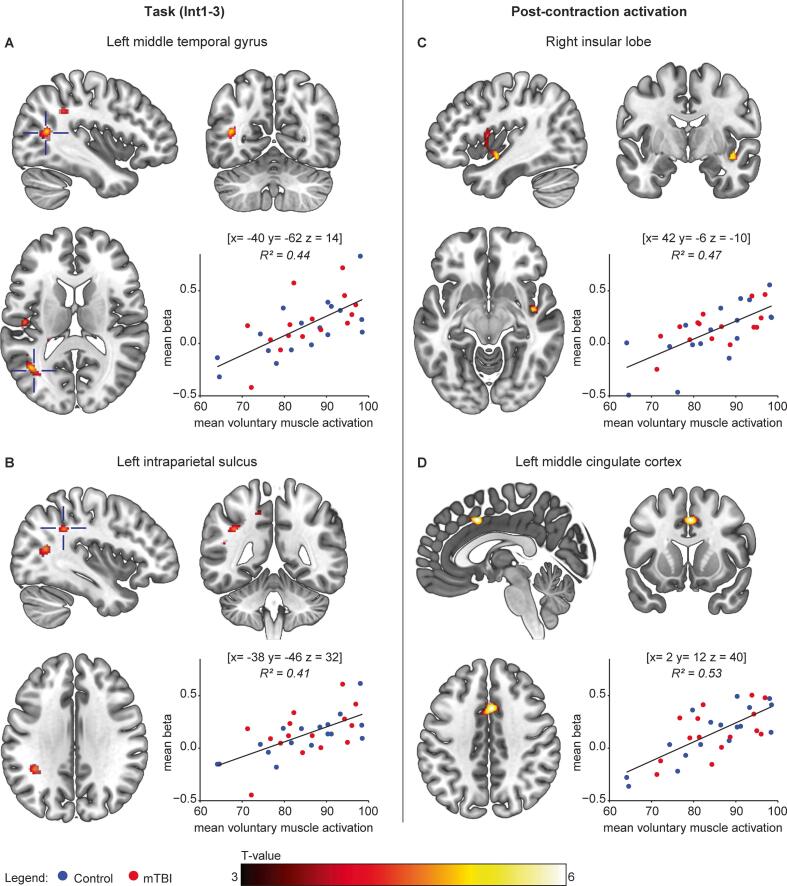
**Associations between voluntary muscle activation and BOLD-activity.***Significant associations were observed between mean voluntary muscle activation during the sustained contraction and BOLD-activity in (A) the left middle temporal gyrus and (B) left intraparietal sulcus. For the post-contraction activation, associations were found in (C) the right insula and (D) the left middle cingulate cortex. For each of the clusters, the relationship between the mean beta’s (entire cluster) and voluntary muscle activation are provided (mTBI = red, control = blue; all p < 0.001). Peak coordinates (MNI space) of the clusters are provided above each plot. A cluster forming threshold of p < 0.001 (uncorrected) was used and all clusters are significant at p < 0.05 (family-wise error corrected), The colour bar indicates voxel T-values, left is left according to neurological convention.* (For interpretation of the references to colour in this figure legend, the reader is referred to the web version of this article.)

Associations between (mean) voluntary muscle activation versus task and post-contraction BOLD-activation were assessed. Clusters showing positive associations with voluntary muscle activation were found for the effect of task (left middle temporal gyrus and intraparietal sulcus), and for post-contraction activation (area Id1 of the right insula and the left middle cingulate cortex; see Fig. 4 and Supplementary Table D). These associations indicate that BOLD-activation was higher in participants who showed better activation of their muscle by the CNS.

### 3.6 BOLD regression (II): self-reported fatigue

**Fig. 5:**
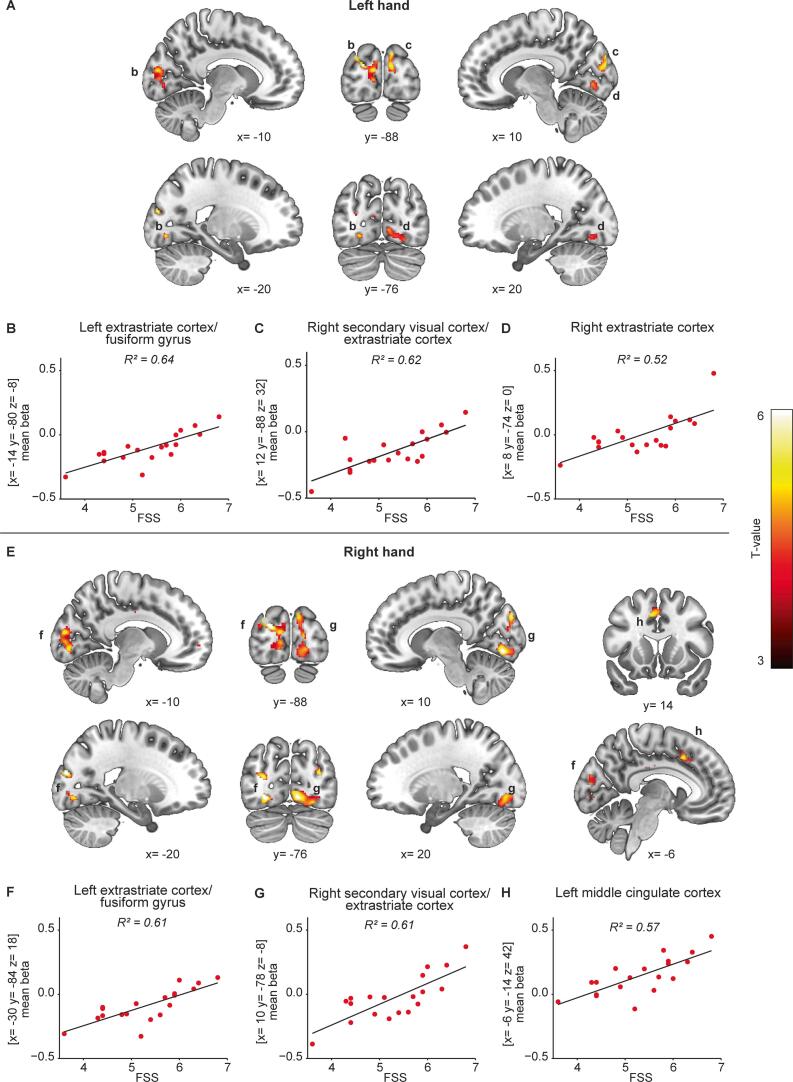
**Associations between self-reported fatigue and BOLD-activity.***In mTBI participants, significant associations were observed between FSS scores (corrected for HADS depression) and BOLD-activity during the submaximal contractions (A) with the left- and (E) right hand. For each of the clusters labelled in A and E, the relationship between the mean beta’s (entire cluster) and FSS scores are provided for the left (B-D) and right hand (F-H). Peak coordinates of the clusters are provided alongside each plot. A cluster forming threshold of p < 0.001 (uncorrected) was used and all clusters are significant at p < 0.05 (family-wise error corrected). All coordinates are shown in MNI space, the colour bar indicates voxel T-values, left is left according to neurological convention.*

Explorative regression analysis was performed in the mTBI group to identify associations between self-reported fatigue (FSS, MFIS_physical_, and MFIS_cognitive_), corrected for HADS depression, and BOLD-activation. Positive associations were observed between the FSS scores and the effect of task for the submaximal contractions. For both hands, associations were observed bilaterally in the visual cortices (mainly extrastriate), and for the right hand in the left middle cingulate. All clusters and correlations are shown in Fig. 5 and Supplementary Table E. No associations between BOLD-activity and the MFIS_physical_ or MFIS_cognitive_ were found, nor for the cognitive test scores.

## 4 Discussion

Our data showed that the mTBI group reported significantly higher levels of self-reported fatigue than the controls. Although no differences in performance fatigability or BOLD-activity were observed between the groups, we found associations between self-reported fatigue (FSS) and BOLD-activation in the left midcingulate cortex and bilateral visual cortices in mTBI participants. Furthermore, across all participants, the level of voluntary muscle activation by the CNS was associated with BOLD-activation in the left middle temporal gyrus and interparietal sulcus during a sustained contraction, but also with long lasting post-contraction activation in the right insula and midcingulate cortex.

### 4.1 mTBI participants report higher levels of fatigue but demonstrate similar levels of performance fatigability.

In line with our previous findings (Prak et al., 2019), most mTBI participants were able to perform the tasks as intended despite increased levels of self-reported fatigue; eighteen out of the nineteen mTBI participants scored above the cut-off of 4 on the FSS questionnaire. During the sustained maximal contraction, two mTBI participants stopped temporarily but were able to continue the task after a few seconds. Although this behaviour suggests that the task was more difficult for the mTBI participants, we did not find any significant differences in task performance between the two groups (conform Prak et al., 2019). Both groups demonstrated performance fatigability as indicated by a decline in voluntary force and force evoked by electrical stimulation of the innervating nerve after the sustained contraction (Enoka and Duchateau, 2016). Additionally, failure of the CNS to sustain maximal drive the muscle, as indicated by a decline in voluntary muscle activation, contributed to the performance fatigability and was similar in mTBI and control groups (Enoka and Duchateau, 2016).

### 4.2 Neural substrate of self-reported fatigue in mTBI

The fact that task performance was similar for the two groups makes interpretation of the BOLD data more straightforward as differences in BOLD-activation were not confounded by differences in task performance (Price and Friston, 1999). Overall, no major differences in BOLD activation were observed between the mTBI and control groups indicating that for the present motor tasks there were no signs of compensatory cortical activation in order to maintain performance. However, we did observe differences within the mTBI group related to the level of self-reported fatigue.

With the submaximal contraction task (task 2), we attempted to modulate effort by increasing force levels. Higher force levels resulted in increased activation of the contralateral sensorimotor cortex and ipsilateral cerebellum on top of the main task effect (Dettmers et al., 1995, van Duinen et al., 2008, Ward and Frackowiak, 2003). Within the mTBI participants associations were found between self-reported fatigue (FFS questionnaire) and activation in visual areas, mainly the extrastriate cortex, as well as the left midcingulate cortex. The activated regions within the extrastriate cortex are involved in motion perception, attention, and oculomotor pursuit (Culham et al., 2001, Jahn et al., 2012, Lencer et al., 2011, Nassi and Callaway, 2009). In our setup, participants matched their force production to visual triggers indicating when and how forcefully they had to contract (see Fig. 1). Monitoring the moving trigger line as well as their own force production can be perceived as an oculomotor pursuit task. Increased activation of higher-order visual areas could therefore be a sign of increased difficulty in attentional related visual processing after mTBI (Ciuffreda et al., 2007, Ventura et al., 2014). Interestingly, in a recent neuroimaging study in multiple sclerosis increased BOLD-activation was observed at a similar location (lingual gyrus and cuneus) in relation to self-reported fatigue and cognitive load (Chen et al., 2020). Furthermore, a relation between the perception of visual effort and (pathological) fatigue was hypothesized in an explanatory model of fatigue by Kuppuswamy (Kuppuswamy, 2021) which fits nicely with our data; higher BOLD-activation related to visual processing associated with increased levels of fatigue. This observation further suggests that visuomotor tasks could be a promising setup to study fatigue in mTBI.

The fatigued mTBI participants also showed increased activation of the left anterior midcingulate cortex. Unfortunately, the fMRI analysis is not so detailed that an exact position within the anterior midcingulate cortex can be discerned. However, the feedback-mediated decision-making model as described by (Bush, 2009, Vogt, 2016) for the dorsal anterior midcingulate cortex (daMCC) is an interesting model to explain our data. This concept presents the function of this part of the MCC as functionally heterogenous neurons which act within cognitive/motor networks to increase the efficiency of decision-making and execution by integrating input from various sources (Vogt, 2016). During a high effort task (e.g., maximal force production), a participant constantly deliberates whether to continue or to stop contracting in the presence of unpleasant feelings related to high effort and muscle soreness. The observation that fatigued mTBI participants showed more BOLD activation in an area involved in feedback-mediated decision-making therefore seems suggestive of increased neuronal activity associated with integration of effort-related afferent inputs. The observation that two of our participants briefly stopped during the sustained contraction may also reflect this constant weighing of options. In our previous studies in individuals with multiple sclerosis (Steens et al., 2012, Wolkorte et al., 2015) or spinal cord injury (Prak et al., 2015) this was seen less often, suggesting this behaviour may be specific to mTBI patients.

The observation that BOLD-activity correlated with the FSS but not the MFIS (physical or cognitive) raises the question which questionnaire is more suitable for indexing fatigue in mTBI. The FSS mainly addresses impact of fatigue on daily activities whereas the MFIS is a multidimensional scale distinguishing physical and cognitive (and also psychosocial) domains (Amtmann et al., 2012, Krupp et al., 1989, Multiple Sclerosis Council for Clinical Practice Guidelines, 1998). Although there is overlap between the FSS and MFIS_physical_, which is also supported by the data of the present study (rho = 0.62, p < 0.01), the MFIS pays more attention to the appraisal of fatigue-related symptoms (Amtmann et al., 2012). The areas in which activation was associated with the FSS scores are areas involved in more complex integrative behaviour and problems integrating various sources of input might be captured by the more generalized FFS questionnaire rather than the MFIS physical or cognitive.

### 4.3 Voluntary muscle activation is associated with increased activity within the effort perception network

The sustained motor task (task 3) was designed to assess time-related changes in BOLD activity in the context of performance fatigability. Over time, a modest increase in activation (Int3 > Int1) was found in a large cluster spanning the left SMA and primary motor cortex, similar to what was previously observed in control participants (Post et al., 2009). This increase in activation probably reflects increased cortical drive necessary to offset use-dependent decline in function in downstream segments of the motor pathway (Post et al., 2009, Taylor et al., 2016). Although BOLD-activation did not differ between mTBI and control participants in the context of performance fatigability, higher levels of voluntary muscle activation during the sustained MVC (i.e., stronger CNS drive) were associated with increased activation in the dorsal part of the left middle temporal gyrus and intraparietal sulcus across both mTBI and control groups. The intraparietal cortex is thought to be involved in sensory-motor integration and generating efference copy signals (Christensen et al., 2007) which makes the increased activation with high voluntary drive understandable.

After the sustained motor task, BOLD-activation in the motor network outlasted the contraction. This prolonged activation after a fatiguing task was seen earlier by our group (Post et al., 2009) and similar prolonged activation was also seen after a fatiguing finger tapping task (Bächinger et al., 2019). By combining fMRI, transcranial magnetic stimulation, and electroencephalography, Bächinger and colleagues (2019) revealed that this post-task activation was mainly due to higher excitation levels within the motor network. The higher excitation being due to reduced intracortical inhibition after fatiguing contractions (but not after non-fatiguing contractions) that showed a relatively slow recovery.

The present data also showed that increased post-contraction activation of the right insula (area Id1) and left midcingulate cortex was associated with stronger CNS drive during the sustained contraction. Thus, the BOLD signal not only demonstrated activity which outlasted the contraction but also differentiated between participants with high and low voluntary drive to the muscle. During contractions, efferent drive also evokes an efference copy, via corollary output, and a stronger output results in increased corollary output. Since both the insula and midcingulate cortex are areas involved in interoception (Beissner et al., 2013, Vogt, 2016), integration of somatosensory information and autonomic regulation, the long-lasting activation of these areas probably reflects increased processing of muscular and somatosensory information related to fatigue-related disturbances in the homeostatic milieu. Higher levels of voluntary activation result in more fatigue-related changes within the muscle fibres as is underlined by the earlier observation that higher voluntary activation is associated with a larger decline in intrinsic muscle force (Prak et al., 2019).

To our knowledge, one other study has utilized a sustained motor paradigm to compare task activation between mTBI and controls (Ramage et al., 2019). In the study by Ramage and colleagues (2019), participants were instructed to self-pace a constant effort for 30 s. While maintaining a constant effort the subject matches sensations evoked by corollary discharges with afferent feedback from active muscles (Monjo et al., 2020). Due to the strong association between perception of effort and perception of fatigue (Jones and Hunter, 1983, Proske and Allen, 2019) it plausible that in this setting the perception of fatigue is also controlled by the subject. This could explain the observed small differences between mTBI and control participants for the main effect of task, but the lack of an effect of time (Ramage et al., 2019).

## 5 Concluding remarks

In conclusion, mTBI participants report increased levels of fatigue compared to controls, but similar task performance. The fMRI data showed that areas related to attentional visual processing show increased activation associated with increased levels of self-reported fatigue in mTBI participants. These results might reflect the importance of sensory effort in fatigued individuals, as advocated by Kuppuswamy (2021). Furthermore, long-lasting cortical activation associated with high voluntary muscle activation might be related to changes in cortical homeostasis in the context of high effort.

## Funding

This work was supported by a grant provided by the Junior Scientific Masterclass of the University Medical Center Groningen to Roeland Prak.

## CRediT authorship contribution statement

**Roeland F. Prak:** Data curation, Formal analysis, Software, Validation, Visualization, Writing – original draft, Writing – review & editing. **Jan-Bernard C. Marsman:** Methodology, Software, Writing – review & editing. **Remco Renken:** Methodology, Software, Writing – review & editing. **Joukje Naalt:** Conceptualization, Writing – review & editing. **Inge Zijdewind:** Conceptualization, Data curation, Formal analysis, Methodology, Software, Supervision, Validation, Writing – original draft, Writing – review & editing.

## Declaration of Competing Interest

The authors declare that they have no known competing financial interests or personal relationships that could have appeared to influence the work reported in this paper.
