# Supplementary material for: Fatigue following mild traumatic brain injury relates to visual processing and effort perception in the context of motor performance
Source: Neuroimage Clin. 2021 Aug 13;32:102783. doi: 10.1016/j.nicl.2021.102783 (PMC8379650; doi:10.1016/j.nicl.2021.102783)
Supplement: Supplementary Data 1 [file mmc1.docx]

**Supplementary Table A.** Summary of the analysis of head motion for the submaximal contraction task. Data are presented as mean (SD). The rms of the realignment parameters (mm) and framewise displacement (mm) are shown for the entire run. The percentage of censored scans (mean, SD) is shown for the entire run and for each individual condition (left hand, right hand). Participants with less than 10 scans remaining in a condition were excluded from the analysis.

|  | rms motion (mm) | | % data censored | | | *n* excluded |
| --- | --- | --- | --- | --- | --- | --- |
|  | raw | FD | total | left hand | right hand |  |
| control (n=19) | 0.49 (0.33) | 0.32 (0.31) | 2.1 (4.3) | 1.5 (3.5) | 0.7 (2.0) | 0 |
| mTBI (n=19) | 0.55 (0.23) | 0.33 (0.13) | 1.5 (1.8) | 1.3 (3.1) | 1.2 (2.7) | 0 |

*rms = root mean square; FD = framewise displacement*

**Supplementary Table B.** Summary of the analysis of head motion for the sustained MVC task. Data are presented as mean (SD). The rms of the realignment parameters (mm) and framewise displacement (mm) are shown for the entire run. The percentage of censored scans (mean, SD) is shown for the entire run and for each individual condition (Int1, Int2, Int3, post-contraction activation). Participants with less than 10 scans remaining in a condition were excluded from the analysis.

|  | rms motion (mm) | | % data censored | | | | | *n* excluded |
| --- | --- | --- | --- | --- | --- | --- | --- | --- |
|  | raw | FD | total | Int1 | Int2 | Int3 | post-contraction |  |
| control (n=19) | 0.63 (0.26) | 0.35 (0.21) | 3.0 (4.7) | 3.6 (6.6) | 8.9 (17.4) | 10.9 (20.50) | 1.6 (3.0) | 2 |
| mTBI (n=19) | 0.65 (0.26) | 0.50 (0.24) | 5.3 (4.7) | 18.1 (17.2) | 20.5 (25.1) | 23.8 (25.8) | 2.3 (2.7) | 3 |

*rms = root mean square; FD = framewise displacement*

**Supplementary Table C.** Regions showing increased BOLD activity at the start (Int1 > Int3) and end (Int3 > Int1) of the sustained MVC. Anatomical regions refer to the entire cluster and are based on the Anatomy Toolbox (Eickhoff et al., 2005) and human motor area template (Mayka et al., 2006). Coordinates (MNI space) and t-values of the peak voxels are provided, as well as cluster p-values (family-wise error corrected) and extent (voxels).

| *Condition* | *Anatomical region* | *Peak coordinates (mm)* | | | *Peak*  *t-value* | *Cluster p-value* | *Extent*  *(voxels)* |
| --- | --- | --- | --- | --- | --- | --- | --- |
|  |  | *x* | *y* | *z* |  |  |  |
| Int1 > Int 3 | Left middle orbital gyrus | -12 | 28 | 10 | 5.11 | 0.000 | 917 |
|  | Right cerebellum (lobule V-VI) | 20 | -52 | -24 | 5.69 | 0.007 | 378 |
|  | Right cerebellum (vermis 4/5) | 2 | -44 | 4 | 4.68 | 0.011 | 340 |
| Int3 > Int1 | Left SMA and M1 | 0 | -14 | 66 | 7.83 | 0.000 | 1038 |

*SMA = supplementary motor area; M1 = primary motor cortex*

**Supplementary Table D.** Regions showing significant (positive) associations with mean voluntary muscle activation during the sustained MVC and BOLD activation *during* (Int1-Int3) and *after* (post-contraction activation) the sustained contraction. Anatomical regions refer to the entire cluster and are based on the Anatomy Toolbox (Eickhoff et al., 2005). Coordinates (MNI space) and t-values of the peak voxels are provided, as well as cluster p-values (family-wise error corrected) and extent (voxels).

| *Condition* | *Anatomical region* | *Peak coordinates (mm)* | | | *Peak*  *t-value* | *Cluster p-value* | *Extent*  *(voxels)* |
| --- | --- | --- | --- | --- | --- | --- | --- |
|  |  | *x* | *y* | *z* |  |  |  |
| Int1-3 | Left middle temporal gyrus | -40 | -62 | 14 | 5.13 | 0.003 | 387 |
|  | Left intraparietal sulcus | -38 | -46 | 32 | 4.82 | 0.026 | 243 |
| Post-contraction activation | Left middle cingulate cortex | 2 | 12 | 40 | 6.20 | 0.019 | 246 |
|  | Right insula | 42 | -6 | -10 | 5.38 | 0.008 | 301 |

**Supplementary Table E.** Regions showing significant association with the FSS questionnaires (corrected for HADS depression) for the submaximal contractions with the left and right hand (effect of task). Anatomical regions refer to the entire cluster and are based on the Anatomy Toolbox (Eickhoff et al., 2005). Coordinates (MNI space) and t-values of the peak voxels are provided, as well as cluster p-values (family-wise error corrected) and extent (voxels).

| *Condition* | *Anatomical region* | *Peak coordinates (mm)* | | | *Peak*  *t-value* | *Cluster p-value* | *Extent*  *(voxels)* |
| --- | --- | --- | --- | --- | --- | --- | --- |
|  |  | *x* | *y* | *z* |  |  |  |
| Task left hand | Left extrastriate cortex/fusiform gyrus | -14 | -80 | -8 | 6.17 | 0.000 | 1025 |
|  | Right secondary visual cortex/extrastriate cortex | 12 | -88 | 32 | 5.68 | 0.016 | 288 |
|  | Right extrastriate cortex | 8 | -74 | 0 | 4.96 | 0.009 | 330 |
| Task right hand | Left extrastriate cortex/fusiform gyrus | -30 | -84 | 18 | 7.37 | 0.000 | 1592 |
|  | Right secondary visual cortex/extrastriate cortex | 10 | -78 | -8 | 6.38 | 0.000 | 1192 |
|  | Left middle cingulate cortex | -6 | 14 | 42 | 5.67 | 0.035 | 236 |
